# Estimation of Visual Function Using Deep Learning From Ultra-Widefield Fundus Images of Eyes With Retinitis Pigmentosa

**DOI:** 10.1001/jamaophthalmol.2022.6393

**Published:** 2023-02-23

**Authors:** Daisuke Nagasato, Takahiro Sogawa, Mao Tanabe, Hitoshi Tabuchi, Shogo Numa, Akio Oishi, Hanako Ohashi Ikeda, Akitaka Tsujikawa, Tadao Maeda, Masayo Takahashi, Nana Ito, Gen Miura, Terumi Shinohara, Mariko Egawa, Yoshinori Mitamura

**Affiliations:** 1 Department of Ophthalmology, Saneikai Tsukazaki Hospital, Himeji, Japan; 2 Department of Ophthalmology, Institute of Biomedical Sciences, Tokushima University Graduate School, Tokushima, Japan; 3 Department of Technology and Design Thinking for Medicine, Hiroshima University Graduate School, Hiroshima, Japan; 4 Department of Ophthalmology and Visual Sciences, Kyoto University Graduate School of Medicine, Kyoto, Japan; 5 Department of Ophthalmology and Visual Sciences, Graduate School of Biomedical Sciences, Nagasaki University, Nagasaki, Japan; 6 Research Center, Kobe City Eye Hospital, Kobe, Japan; 7 Laboratory for Retinal Regeneration, RIKEN Center for Biosystems Dynamics Research, Kobe, Japan; 8 Vision Care Inc, Kobe, Japan; 9 Department of Ophthalmology and Visual Science, Chiba University Graduate School of Medicine, Chiba, Japan

## Abstract

**Importance:**

There is no widespread effective treatment to halt the progression of retinitis pigmentosa. Consequently, adequate assessment and estimation of residual visual function are important clinically.

**Objective:**

To examine whether deep learning can accurately estimate the visual function of patients with retinitis pigmentosa by using ultra-widefield fundus images obtained on concurrent visits.

**Design, Setting, and Participants:**

Data for this multicenter, retrospective, cross-sectional study were collected between January 1, 2012, and December 31, 2018. This study included 695 consecutive patients with retinitis pigmentosa who were examined at 5 institutions. Each of the 3 types of input images—ultra-widefield pseudocolor images, ultra-widefield fundus autofluorescence images, and both ultra-widefield pseudocolor and fundus autofluorescence images—was paired with 1 of the 31 types of ensemble models constructed from 5 deep learning models (Visual Geometry Group–16, Residual Network–50, InceptionV3, DenseNet121, and EfficientNetB0). We used 848, 212, and 214 images for the training, validation, and testing data, respectively. All data from 1 institution were used for the independent testing data. Data analysis was performed from June 7, 2021, to December 5, 2022.

**Main Outcomes and Measures:**

The mean deviation on the Humphrey field analyzer, central retinal sensitivity, and best-corrected visual acuity were estimated. The image type–ensemble model combination that yielded the smallest mean absolute error was defined as the model with the best estimation accuracy. After removal of the bias of including both eyes with the generalized linear mixed model, correlations between the actual values of the testing data and the estimated values by the best accuracy model were examined by calculating standardized regression coefficients and *P* values.

**Results:**

The study included 1274 eyes of 695 patients. A total of 385 patients were female (55.4%), and the mean (SD) age was 53.9 (17.2) years. Among the 3 types of images, the model using ultra-widefield fundus autofluorescence images alone provided the best estimation accuracy for mean deviation, central sensitivity, and visual acuity. Standardized regression coefficients were 0.684 (95% CI, 0.567-0.802) for the mean deviation estimation, 0.697 (95% CI, 0.590-0.804) for the central sensitivity estimation, and 0.309 (95% CI, 0.187-0.430) for the visual acuity estimation (all *P* < .001).

**Conclusions and Relevance:**

Results of this study suggest that the visual function estimation in patients with retinitis pigmentosa from ultra-widefield fundus autofluorescence images using deep learning might help assess disease progression objectively. Findings also suggest that deep learning models might monitor the progression of retinitis pigmentosa efficiently during follow-up.

## Key Points

**Question:**

Can artificial intelligence estimate the visual function of eyes with retinitis pigmentosa from ultra-widefield fundus images?

**Findings:**

In this multicenter cross-sectional study of 1274 eyes of 695 patients with retinitis pigmentosa, the standardized regression coefficient was 0.309 in estimating visual acuity based on ultra-widefield fundus autofluorescence images using a deep learning model, 0.684 in estimating the mean deviation on the Humphrey field analyzer, and 0.697 in estimating central retinal sensitivity.

**Meaning:**

Findings suggest that this estimation method of visual function using artificial intelligence with ultra-widefield fundus autofluorescence images assists in objectively evaluating the progression of retinitis pigmentosa.

## Teaser

This cross-sectional study investigates whether deep learning can estimate the visual function of eyes with retinitis pigmentosa from ultra-widefield fundus images.

## Introduction

Retinitis pigmentosa (RP) is associated with many photoreceptor-specific gene variations. Thus, the degeneration of photoreceptor cells presents different progressive patterns.^1,2^ Although new treatments for RP are being developed,^3,4,5^ current practice mainly involves care for residual visual function and surgery or medical therapy for complications. Hence, an appropriate clinical evaluation and estimation method for residual visual function in patients with RP should be established.

Fundus autofluorescence (FAF) reflects retinal pigment epithelium functions by visualizing the accumulation of lipofuscin.^6^ Fundus autofluorescence images of patients with RP show hyperfluorescence in the early disease stages, whereas hypofluorescence corresponds to lesions in later stages. In typical RP, an AF ring, which represents a hyperfluorescent ring in FAF images, might be observed at the border separating the dysfunctional from the functional retina.^7,8^ Ultra-widefield pseudocolor (UWPC) and ultra-widefield FAF (UWFAF) imaging using a scanning laser ophthalmoscope enables clinicians to obtain fundus images with a 200° angle of view both easily and noninvasively. Several studies have reported correlations between the findings of FAF, such as AF rings in RP, and each finding of the following techniques: Goldmann perimetry, Humphrey field analyzer (HFA), and optical coherence tomography (OCT).^9,10,11,12,13,14,15^

In recent years, image-processing approaches using deep learning (DL) models have been applied to various diagnostic imaging applications.^16,17,18,19,20^ Previously, we reported several applications of image-processing technologies using DL in ophthalmology.^21,22,23,24,25,26^ However, we believe that there are few applications of image-processing technology using DL models to quantitatively estimate visual function in RP. In this study, we investigated whether DL models can estimate visual function in patients with RP by using ultra-widefield fundus images obtained on concurrent visits.

## Methods

### Study Design and Overview

This retrospective, multicenter, cross-sectional study was conducted from January 1, 2012, to December 31, 2018, according to the Declaration of Helsinki^27^ and was approved by the institutional review boards of Saneikai Tsukazaki Hospital, Tokushima University, Kyoto University, Chiba University, and Kobe City Eye Hospital, Japan; and written informed consent without incentive was obtained from all patients. This study followed the Strengthening the Reporting of Observational Studies in Epidemiology (STROBE) reporting guideline.

We retrieved the UWPC and UWFAF images and clinical data of consecutive patients with RP from the clinical databases of the 5 mentioned institutions. We diagnosed RP based on patient clinical findings and results from fluorescein angiography and full-field electroretinograms with the recording protocol conforming to the International Society for Clinical Electrophysiology of Vision standards.^28^ All patients with RP showed distinctive fundus findings, such as retinal vessel constriction, optic disc atrophy, bone-spicule pigment clumping, and rod-cone dystrophy, detected with electroretinography. We excluded patients with atypical RP, such as unilateral or sector RP, and those with uveitis or other conditions that can present with fundus findings similar to RP. Eyes with dense cataracts that precluded ultra-widefield scanning laser ophthalmoscope examinations, macular edema, epiretinal membrane, or myopia with posterior staphyloma were also excluded. A total of 1274 images obtained from the 1274 eyes of 695 patients with RP were studied, 1 image of each eye. The presence or absence of AF rings on UWFAF images was confirmed. The presence of an AF ring was determined independently by 2 authors (T. Sogawa and T. Shinohara), for whom the clinical data were masked. In the case of disagreement, another author (M.E.) joined the discussion and assisted with the final decision. In this context, 757 eyes from 419 patients had the AF ring, whereas 517 eyes from 304 patients were without it.

We obtained UWPC and UWFAF images from the “all-eyes” group by using an ultra-widefield scanning laser ophthalmoscope (Optos 200Tx; Optos PLC), and we measured the best-corrected visual acuity (BCVA), mean deviation (MD), and mean sensitivity of central 12 test points (CENT12) using the HFA 10-2 program (Carl Zeiss Meditec AG). The BCVA was measured with a standard Japanese Landolt visual acuity chart at a test distance of 5 m, was corrected on the basis of the subjective and objective refraction test results, and was converted into logMAR units. We used the Swedish interactive threshold algorithm standard’s testing algorithm in the HFA measurement. All ophthalmologic examinations were performed on the same day. The all-eyes data set included 3 types of input images: UWPC, UWFAF, and both UWPC and UWFAF images. The DL model estimated MD, CENT12, and BCVA in the all-eyes group.

To examine whether the presence or absence of AF rings was associated with the estimation accuracy of the DL model, we prepared the data sets of the presence or absence of AF rings by using only UWFAF images. Thereafter, we analyzed a total of 6 patterns in which the DL model estimated MD, CENT12, and BCVA in both groups, AF rings present or absent.

### DL Model and Its Training

To construct ensemble models, we used the following 5 DL models: Visual Geometry Group–16, Residual Network–50, InceptionV3, DenseNet121, and EfficientNetB0. Because there were 31 combinations to build an ensemble model consisting of 1 to 5 models using these 5 DL models, 31 different ensemble models were constructed. After training these models, we evaluated the performance of each one.^29,30,31,32,33^ In the all-eyes group, we used 848, 212, and 214 images of the data from patients for the training, validation, and testing data, respectively. In the groups with AF rings present or absent, we used 502 and 343 images for the training data, 126 and 86 images for the validation data, and 129 and 88 images for the testing data, respectively. The splits of data were done at the patient level. All data from Chiba University were used for the independent testing data but not for training or validation.

The aspect ratio of the original ultra-widefield fundus images was 3900 × 3072 pixels. A study reported that the performance of a DL model depends on the image resolution and cropping range.^34^ Our preliminary study indicated that the estimation accuracy was higher for images of 512 × 512 pixels without cropping (eAppendix, eFigure 1, eTable 1, and eTable 2 in Supplement 1). Therefore, further analyses were performed after image resizing to 512 × 512 pixels without cropping. Because the RGB (red, green, and blue) image input ranges from 0 to 255, we divided the value by 255 and normalized it to a range of 0 to 1. Then we trained the deep neural network by using data augmentation techniques for each epoch, such as brightness adjustment, gamma correction, and noise addition.^21,22,23^ For the estimation of both UWPC and UWFAF images, we used 2 image modalities passing through different convolutional neural networks and added a network that combined them.

In this network training process, we used the same process as in previous reports,^26,29,30,31,35,36^ up to the flattening process. For the remaining processes, we performed global mean pooling in 2 dimensions and converted the data to 1 dimension. The obtained data were then compressed to 256 units by using a fully connected layer, which we used again to output 1 value. In the concatenated network, each of the input UWFAF and UWPC images was compressed into 256 units of features and concatenated. Then they passed through a 64-unit, fully connected layer and output as a single estimation.

As for the transfer learning, we performed fine-tuning or fully retraining using the parameters of the model that learned the ImageNet data set (Stanford Vision Lab) as the initial values for the layers before the flattening process. This step enabled the network to achieve high performance even with a small amount of data.^37^ We performed model training and validation with Keras, an application programming interface of Python TensorFlow.^38^

### Statistical Analysis

For each of the 3 image types of the all-eyes group, we estimated the visual functions with the 31 different kinds of DL ensemble models. Then we evaluated the performance of estimation on the visual function for 93 types of total image-model combinations, which were 93 patterns obtained by combining 31 ensemble models with 3 types of images. Similarly, we used these 31 DL models to estimate the visual function from the UWFAF images alone in the groups with AF rings present or absent. When the DL models were combined to construct an ensemble model, the mean of each model’s output was used as the output of the ensemble model. Validation data were used to determine which ensemble model was optimal. We calculated the mean absolute error (MAE), the root-mean-square error (RMSE), and the Pearson correlation coefficient and compared the magnitude of errors between the estimated values and the actual values for each image-model combination. We used the MAE as the performance metric and adopted the image-model combination with the smallest MAE as the combination with the best estimation accuracy.

The MAE and RMSE are common metrics used to compare the accuracy of machine learning models.^39,40^ Nevertheless, according to several artificial intelligence (AI) studies,^39,41,42^ we used MAE as a basis for selecting the best model in our study because, compared with RMSE, it is less sensitive to outliers and has a better metric for selecting the best model among the ensemble models.^43^

For the image-model combination with the smallest MAE, we computed standardized regression coefficients (SRCs) of estimated and actual values in the testing data. For regression analysis, we used the restricted maximum likelihood approach.^44^ To eliminate bias due to the inclusion of both eyes of a patient in the analysis, we constructed generalized linear mixed models for the SRC, its 95% CIs, and *P* values, as follows: pred′[n] ~ Normal (α[PID[n]] + β × actual′[n], σ*_p_*), α[*k*] = α_all_ + α*_id_*[*k*], α*_id_*[*k*] ~ Normal (0, σ_α_), where pred′ is an estimated value that is standardized so that it has a mean of 0 and a variance of 1, actual′ is an actual value processed in the same way, PID is a variable that stores which data belong to which patient, Normal indicates normal distribution, σ indicates SD, α indicates intercept, and β indicates slope. The *P* values were 2-sided and not adjusted for multiple analyses. We used Statsmodels (Statsmodels developers) version 0.13.5,^45^ a Python package, for SRC analysis and SciPy (version 1.7.3),^46^ another Python library, for statistical analyses other than SRC. Data analysis was performed from June 7, 2021, to December 5, 2022.

### Heat Map

We generated a heat map to illustrate where the 5 DL models focused on UWFAF images to estimate MD. We used the Score-CAM method to create the heat map^47^ and the ReLU function to correct the loss function during back-propagation. The target layer was the final convolutional layer of the fifth block. We used tf-keras-vis, version 0.8.2 (Keisen)^48^ a Python package, to create heat maps.

## Results

A total of 1668 eyes of 856 Japanese patients with RP were consecutively extracted from the clinical databases. After the exclusion criteria were applied, 1274 eyes of 695 patients were included in this study. A total of 310 patients were male (44.6%), 385 were female (55.4%), and the mean (SD) age was 53.9 (17.2) years. Table 1 presents the clinical characteristics of the all-eyes group with AF rings present or absent. The mean age was higher in the group without AF rings than with AF rings (mean [SD] age, 55.5 [16.9] years vs 52.8 [17.4] years; difference, 2.7 [95% CI, 0.8-4.6]; *P* = .005), whereas the MD, CENT12, and BCVA in the group without AF rings were worse than those of the group with AF rings (mean [SD]: MD, −22.7 [10.1] vs −14.5 [9.6]; difference, −8.2 [95% CI, −9.3 to −7.1]; CENT12, 14.8 [10.8] vs 25.2 [8.7]; difference, −10.4 [95%CI, −11.5 to −9.2]; BCVA, 0.55 [0.61] vs 0.22 [0.45]; difference, 0.33 [95% CI, 0.26-0.39]; all *P* < .001).

**Table 1.** Clinical Characteristics of the Participants With Positive AF Ring and Those With Negative AF Ring

Characteristic	All eyes	AF ring	
		Positive	Negative
No. of eyes	1274	757	517
Age, mean (SD), y	53.9 (17.2)	52.8 (17.4)	55.5 (16.9)
Sex, No. (%)			
Male	551 (43.2)	322 (42.5)	229 (44.3)
Female	723 (56.8)	435 (57.5)	288 (55.7)
Laterality, left, No. (%)	634 (49.8)	377 (49.8)	257 (49.7)
MD, mean (SD), dB	−17.8 (10.6)	−14.5 (9.6)	−22.7 (10.1)
CENT12, mean (SD), dB	21.0 (10.9)	25.2 (8.7)	14.8 (10.8)
BCVA, mean (SD), logMAR	0.36 (0.55)	0.22 (0.45)	0.55 (0.61)

Abbreviations: AF, autofluorescent; BCVA, best-corrected visual acuity; CENT12, mean sensitivity of central 12 test points on the Humphrey field analyzer; MD, mean deviation on the Humphrey field analyzer.

**Figure 1.**
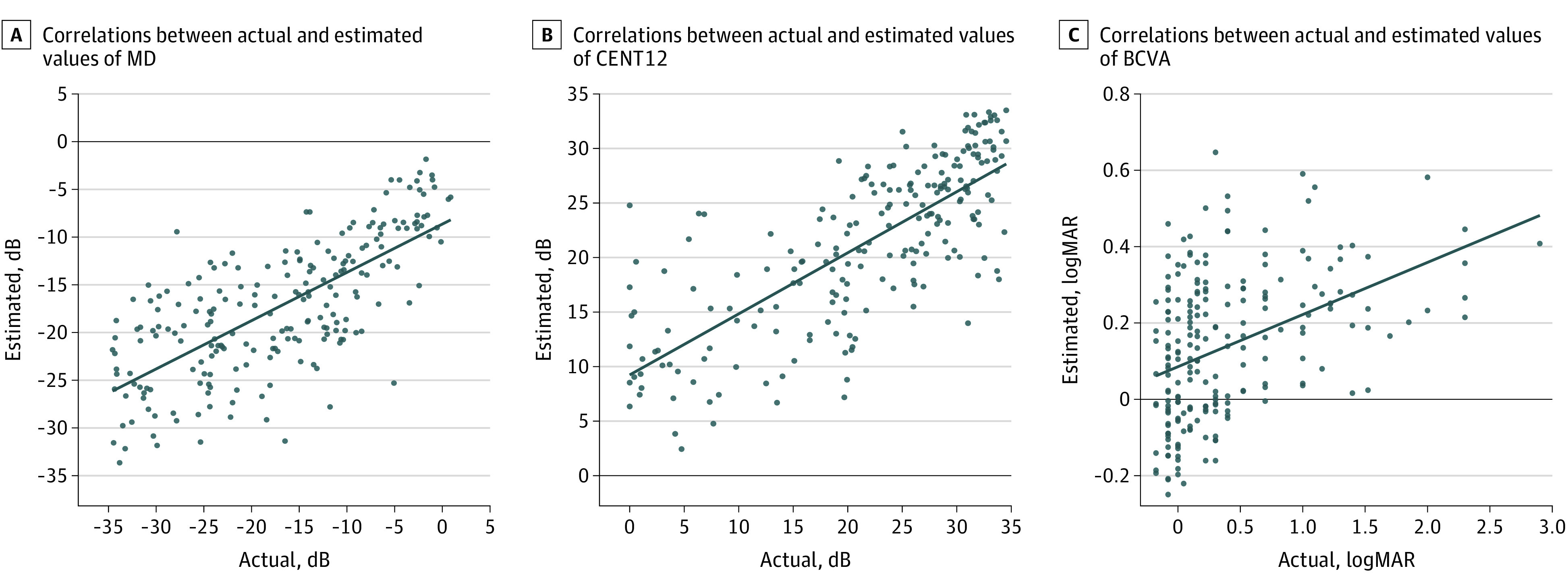
Correlations Between Actual Values of Visual Function and Estimated Values by Deep Learning Model Correlations pertain to the all-eyes group. BCVA indicates best-corrected visual acuity; CENT12, mean sensitivity of central 12 test points on the Humphrey field analyzer; and MD, mean deviation on the Humphrey field analyzer.

The image type in which the model yielded the smallest MAE value in the all-eyes group was the UWFAF image alone for estimating MD, CENT12, and BCVA (Table 2). As for the DL models, the ensemble model with the best estimation accuracy comprised EfficientNetB0 and InceptionV3, InceptionV3 and Visual Geometry Group–16, and EfficientNetB0 and Visual Geometry Group–16 for estimating MD, CENT12, and BCVA, respectively. The SRC was 0.684 (95% CI, 0.567-0.802; *P* < .001) for MD estimation, 0.697 (95% CI, 0.590-0.804; *P* < .001) for CENT12 estimation, and 0.309 (95% CI, 0.187-0.430; *P* < .001) for BCVA estimation (Table 2; Figure 1).

**Table 2.** Correlations Between Actual Values of Visual Function and Estimated Values by Deep Learning Model^a^

Data set and parameter	Image	DL model	Correlation coefficient	SRC (95% CI)	*P* value
All eyes					
MD, dB	UWFAF	EfficientNetB0, InceptionV3	0.715	0.684 (0.567 to 0.802)	<.001
CENT12, dB	UWFAF	InceptionV3, VGG16	0.757	0.697 (0.590 to 0.804)	<.001
BCVA, logMAR	UWFAF	EfficientNetB0, VGG16	0.417	0.309 (0.187 to 0.430)	<.001
MD, dB					
AF ring positive	UWFAF	ResNet50, VGG16	0.680	0.568 (0.398 to 0.738)	<.001
AF ring negative	UWFAF	DenseNet121	0.521	0.274 (0.030 to 0.518)	.03
CENT12, dB					
AF ring positive	UWFAF	ResNet50, VGG16	0.731	0.660 (0.513 to 0.807)	<.001
AF ring negative	UWFAF	EfficientNetB0	0.168	0.186 (−0.025 to 0.396)	.08
BCVA, logMAR					
AF ring positive	UWFAF	DenseNet121, EfficientNetB0	0.311	0.279 (0.086 to 0.472)	.005
AF ring negative	UWFAF	InceptionV3, ResNet50, VGG16	0.217	0.094 (−0.082 to 0.270)	.30

Abbreviations: AF, autofluorescent; BCVA, best-corrected visual acuity; CENT12, mean sensitivity of central 12 test points on the Humphrey field analyzer; DL, deep learning; MD, mean deviation on the Humphrey field analyzer; ResNet50, Residual Network–50; SRC, standardized regression coefficient; UWFAF, ultra-widefield fundus autofluorescence; VGG16, Visual Geometry Group–16.

^a^ The image–DL model combination with the smallest mean absolute error is displayed.

The data sets for the presence or absence of AF rings were used to examine whether the presence or absence of AF rings in UWFAF images affected the estimation accuracy of DL models. For estimations in the group with the presence of AF rings, the SRC values for estimating MD, CENT12, and BCVA were 0.568 (95% CI, 0.398-0.738; *P* < .001), 0.660 (95% CI, 0.513-0.807; *P* < .001), and 0.279 (95% CI, 0.086-0.472; *P* = .005) (Table 2 and eFigure 2 in Supplement 1), respectively. The SRC values for estimating MD, CENT12, and BCVA in the group without the presence of AF rings were 0.274 (95% CI, 0.030-0.518; *P* = .03), 0.186 (95% CI, −0.025 to 0.396; *P* = .08), and 0.094 (95% CI, −0.082 to 0.270; *P* = .30), respectively. When the model used the data set for the presence of AF rings, the estimation accuracy for MD, CENT12, and BCVA improved compared with that using the data set for the absence of AF rings. The MAE and RMSE for each model are presented in eTable 3 in Supplement 1.

**Figure 2.**
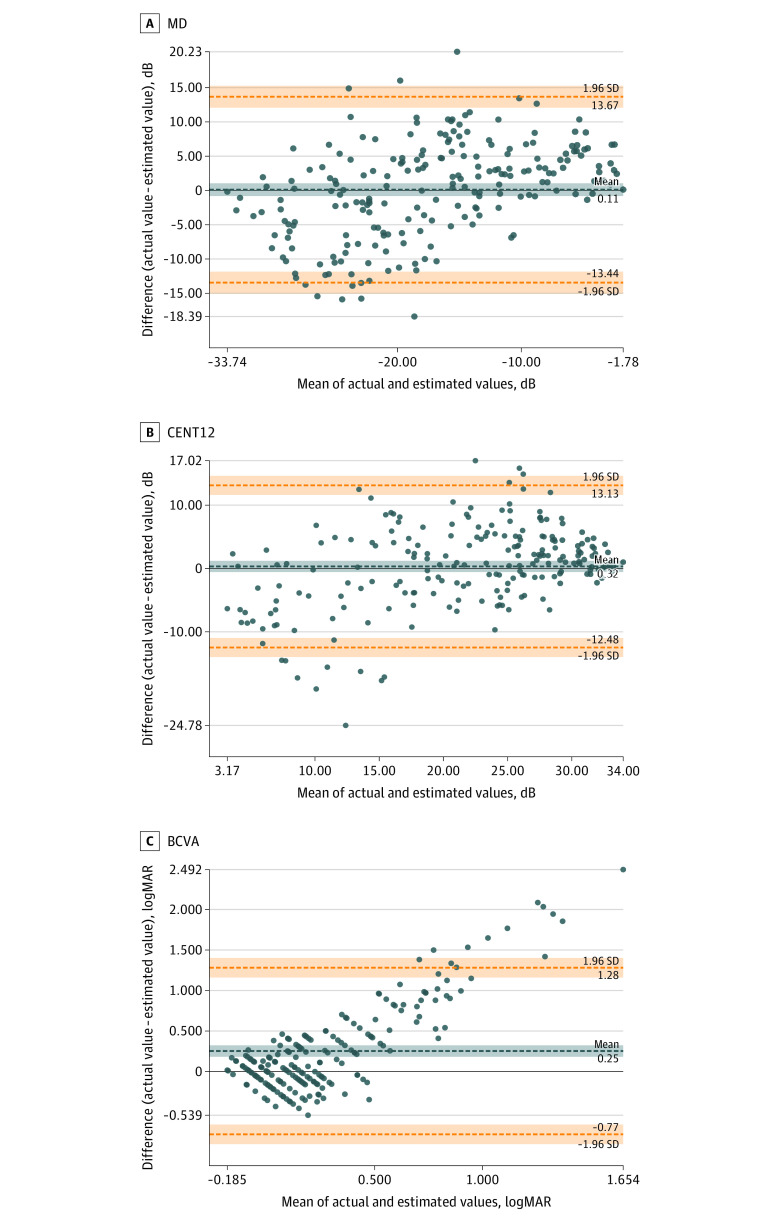
Bland-Altman Plots of Visual Function Between Actual Values and Estimated Values by Deep Learning Bland-Altman plots pertain to the all-eyes group. More than 95% of the differences in MD values lie within the limits of agreement. However, proportional bias was prominent in BCVA and existed in MD (correlation coefficient, 0.45; 95% CI, 0.34-0.55; *P* < .001), CENT12 (correlation coefficient, 0.42; 95% CI, 0.31-0.53; *P* < .001), and BCVA (correlation coefficient, 0.83; 95% CI, 0.79-0.87; *P* < .001). There was no fixed bias in MD (mean difference, 0.11; 95% CI, −0.82 to 1.05; *P* = .81) or CENT12 (mean difference, 0.32; 95% CI, −0.56 to 1.21; *P* = .47), whereas there was a fixed bias in BCVA (mean difference, 0.25; 95% CI, 0.18-0.32; *P* < .001). BCVA indicates best-corrected visual acuity; CENT12, mean sensitivity of central 12 test points on the Humphrey field analyzer; and MD, mean deviation on the Humphrey field analyzer.

Bland-Altman plots are presented in Figure 2 and eFigure 3 in Supplement 1. In the all-eyes group, there was a fixed bias in BCVA but not in MD or in CENT12. Proportional bias existed in all 3 parameters and was prominent in the BCVA.

**Figure 3.**
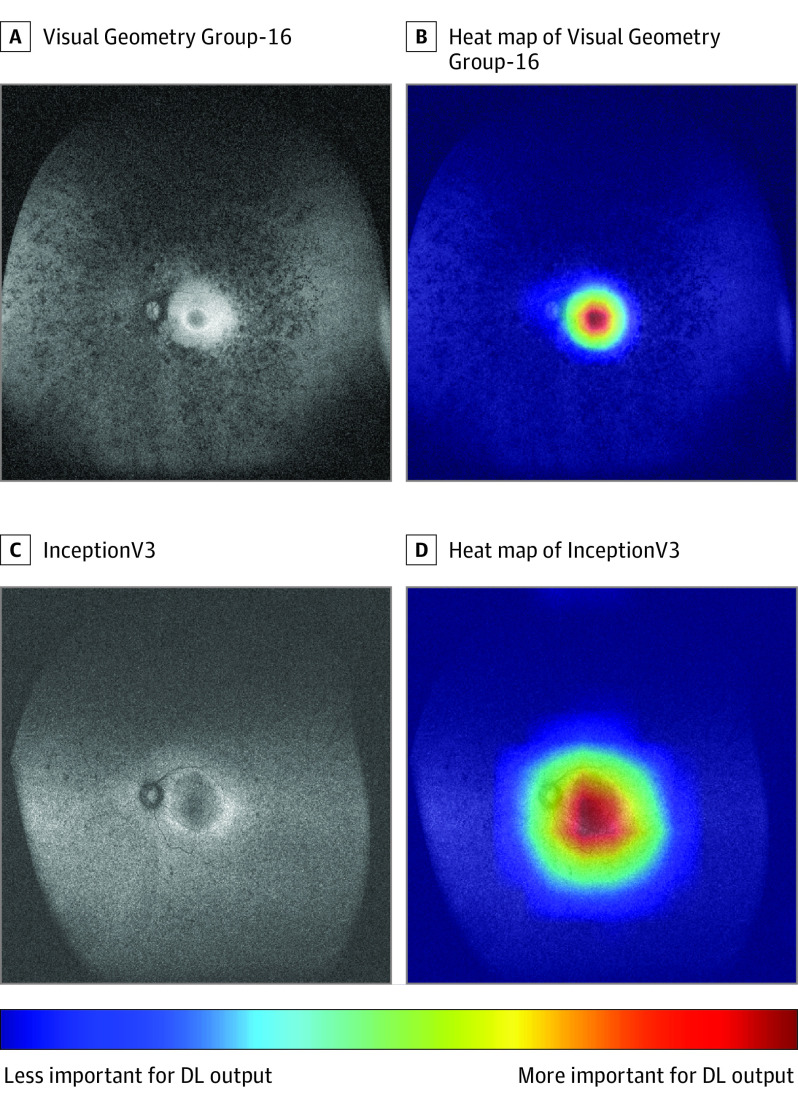
Representative Ultra-Widefield Fundus Autofluorescence (UWFAF) Images and Their Heat Map Images When the mean deviation on the Humphrey field analyzer was estimated using deep learning (DL) models of Visual Geometry Group–16 (A and B) and InceptionV3 (C and D), heat map images were superimposed on the UWFAF images in the composite images.

Figure 3 and eFigure 4 in Supplement 1 show typical UWFAF images and composite images of heat maps superimposed on the UWFAF images. The areas around the fovea, AF ring, and margins of the degenerated retina are shown in warm colors, indicating the areas where the 5 DL models focused when estimating the MD values.

## Discussion

In this study, we found correlations between the actual values of MD, CENT12, and BCVA and the values estimated by the DL model from UWFAF images, although correlation and consistency were slightly weak in the BCVA estimation. These results are consistent with previous reports showing high correlation between HFA parameters or BCVA and the radius or internal area of the AF ring on FAF images.^9,12,49^ Previously, we reported the capability of the DL model to distinguish between RP images and normal fundus images with high sensitivity and specificity, using UWPC and UWFAF images.^21^ The present study represents the findings that proved the capability of the DL model to evaluate quantitatively the residual visual function of patients with RP from UWFAF images. Because UWFAF images can be obtained easily, quickly, and noninvasively without mydriasis, the ability to estimate the visual functions in patients with RP from these images would be an additional benefit in routine clinical practice. This might indicate that obtaining UWFAF images would enable ophthalmologists to monitor RP progression during a follow-up period.

Our data showed that the estimation accuracy of the DL model tends to be higher when the estimation was made from UWFAF images alone, contrary to the ones made either from UWPC images alone or from both UWPC and UWFAF images. Deep learning models can learn complex, individualized local features in image data and build optimal structures to identify those features.^50,51,52^ The DL model had higher estimation accuracy from UWFAF images alone likely because this type of image had more information for the model. Thus, the information on the retinal pigment epithelium function reflected in the FAF images could be highly beneficial in estimating visual functions.

In RP, the hyperfluorescent area of an AF ring on FAF images, which is considered to indicate increased phagocytosis of the photoreceptor outer segment, is found at the same location as the disappearance of the ellipsoid zone on OCT images.^12,53,54,55^ Thus, the AF ring indicates the border of the retinal impairment. Inoue et al^56^ used semiautomatic software to estimate the retinal sensitivity from age, BCVA, and FAF images in 93 eyes with RP and found that the estimation accuracy was higher for images with AF rings than for images without AF rings. Consistent with these results, estimating the visual function using only images with the AF rings showed better SRC in this study.

As described earlier, visual function is related closely to OCT and FAF findings. Therefore, the DL model can estimate visual function adequately from the UWFAF images. Most recently, a study reported that a binary classification of better or worse than a BCVA of 20/40 could be estimated by the DL model with OCT and infrared images.^57^ However, a previous study determined that the AF ring area on FAF images is more closely related to retinal sensitivity than the area of ellipsoid zone loss on OCT images.^58^ In addition, the UWFAF images used in this study can capture the degenerative process of the photoreceptor and retinal pigment epithelium cells in RP over a wider area than OCT images, which indicates that UWFAF images are more useful.

A major concern regarding the FAF findings in earlier studies is that the AF ring location was determined by humans; hence, it was not assessed objectively. To evaluate FAF images objectively, analyses by AI appear to be useful. The concern regarding AI analyses is that the essential lesion sites on medical images, on which AI focuses, might not be the same as the sites where ophthalmologists look when determining a diagnosis or evaluating visual function. However, in this study, the heat maps showed a warm color around the fovea, AF ring, and margins of the degenerated retina. This indicates that the areas where the DL model focuses are consistent with the areas where ophthalmologists focus when judging the severity of visual impairment from fundus images. Therefore, these heat map data suggest that the DL model accurately identifies the degenerated area of the RP and estimates the visual function according to the degeneration features.

### Limitations

This study has several limitations. First, the progression patterns of RP vary widely.^59,60^ Therefore, whether the DL model can estimate the visual function in patients with RP that deviates from the progression patterns of RP analyzed here is unclear. Second, because this was a retrospective study, the selection of images included here might be biased. Third, the Early Treatment Diabetic Retinopathy Study visual acuity chart was not used for the BCVA measurement. Fourth, it is intrinsically unclear what the DL model identifies to estimate the visual function.

## Conclusions

The results of this study reveal correlations between the actual values of the visual function and the estimated values by the DL model using UWFAF images. The estimation of visual function using DL for patients with RP might help clinicians assess the progression of RP objectively.

## Notes

Supplement 1. **eAppendix.** Preliminary Study for Determining Image Cropping and Resolution **eTable 1.** Estimation Accuracy for Mean Deviation Using Ultra-widefield Fundus Autofluorescence (UWFAF) Images With and Without Cropping **eTable 2.** Estimation Accuracy for Mean Deviation Using Ultra-widefield Fundus Autofluorescence Images With Different Image Resolutions **eTable 3.** Mean Absolute Error (MAE) and Root-Mean-Square Error (RMSE) **eFigure 1.** Cropping of the Ultra-widefield Fundus Autofluorescence Image of the Representative Case **eFigure 2.** Correlations Between Actual Values and Estimated Values in Groups With the Presence or Absence of AF Rings **eFigure 3.** Bland-Altman Plots Between Actual Values and Estimated Values in Groups With the Presence or Absence of AF Rings **eFigure 4.** Representative Ultra-widefield Fundus Autofluorescence (UWFAF) Images and Their Heat Map Images

Supplement 2.
Data Sharing Statement
