# Supplementary material for: Estimation of Visual Function Using Deep Learning From Ultra-Widefield Fundus Images of Eyes With Retinitis Pigmentosa
Source: JAMA Ophthalmol. 2023 Feb 23;141(4):305–13. doi: 10.1001/jamaophthalmol.2022.6393 (PMC9951103; doi:10.1001/jamaophthalmol.2022.6393)
Supplement: Supplement 1. — eAppendix. Preliminary Study for Determining Image Cropping and Resolution eTable 1. Estimation Accuracy for Mean Deviation Using Ultra-widefield Fundus Autofluorescence (UWFAF) Images With and Without Cropping eTable 2. Estimation Accuracy for Mean Deviation Using Ultra-widefield Fundus Autofluorescence Images With Different Image Resolutions eTable 3. Mean Absolute Error (MAE) and Root-Mean-Square Error (RMSE) eFigure 1. Cropping of the Ultra-widefield Fundus Autofluorescence Image of the Representative Case eFigure 2. Correlations Between Actual Values and Estimated Values in Groups With the Presence or Absence of AF Rings eFigure 3. Bland-Altman Plots Between Actual Values and Estimated Values in Groups With the Presence or Absence of AF Rings eFigure 4. Representative Ultra-widefield Fundus Autofluorescence (UWFAF) Images and Their Heat Map Images [file jamaophthalmol-e226393-s001.pdf]

Supplementary Online Content

Nagasato D, Sogawa T, Tanabe M, et al. Estimation of visual function using deep learning from ultra-widefield fundus images of eyes with retinitis pigmentosa. *JAMA Ophthalmol.* Published online February 23, 2023. doi:10.1001/jamaophthalmol.2022.6393

eAppendix. Preliminary Study for Determining Image Cropping and Resolution

eTable 1. Estimation Accuracy for Mean Deviation Using Ultra-widefield Fundus Autofluorescence (UWFAF) Images With and Without Cropping

eTable 2. Estimation Accuracy for Mean Deviation Using Ultra-widefield Fundus Autofluorescence Images With Different Image Resolutions

eTable 3. Mean Absolute Error (MAE) and Root-Mean-Square Error (RMSE)

eFigure 1. Cropping of the Ultra-widefield Fundus Autofluorescence Image of the Representative Case

eFigure 2. Correlations Between Actual Values and Estimated Values in Groups With the Presence or Absence of AF Rings

eFigure 3. Bland-Altman Plots Between Actual Values and Estimated Values in Groups With the Presence or Absence of AF Rings

eFigure 4. Representative Ultra-widefield Fundus Autofluorescence (UWFAF) Images and Their Heat Map Images

This supplementary material has been provided by the authors to give readers additional information about their work.

© 2023 Nagasato D et al. *JAMA Ophthalmology.*

eAppendix. Preliminary Study for Determining Image Cropping and Resolution

First, we performed a preliminary study to investigate the optimal image-cropping methods and resolution of the analyzed images.

Cropping of the images

We initially investigated how the estimation accuracy changes according to the cropping of images. When estimating the mean deviation (MD) value in the all-eyes group with the five deep learning (DL) models (Visual Geometry Group-16, Residual Network-50, InceptionV3, DenseNet121, and EfficientNetB0), we compared the estimation accuracy between ultra-widefield fundus autofluorescence (UWFAF) images with and without cropping. The aspect ratio of the original ultra-widefield fundus images was 3900 × 3072 pixels. Although the optimal resolution of images was unknown at this point, the fundus images were tentatively resized to 512 × 512 pixels. By cropping, we automatically excluded the surrounding black portion (eFigure 1). The cropping area was specified as the largest rectangular area containing the optic disc, macula, and fundus, with no surrounding black area. As a result, the estimation accuracy using the validation data, which was evaluated by correlation coefficient, was higher for images without cropping (eTable 1). Therefore, all further analyses in this study were performed without image cropping.

Resolution of images

In the next stage, we investigated the optimal resolution of the analyzed images. When reducing the image resolution, the advantages of the ultra-widefield fundus images and many information may be lost. However, as the image resolution increases, the analysis time increases exponentially, and there is also the limit of Graphics Processing Unit memory. Therefore, to determine the optimal image resolution, we initially compared the estimation accuracy between 512 × 512-pixel and 1024 × 1024-pixel images. When MD estimation was performed using UWFAF images in the all-eyes group with the five DL models, the estimation accuracy was ameliorated by 512 × 512-pixel images in four kinds of DL models, except for the InceptionV3 (eTable 2). Based on these results, we considered that 512 × 512 pixels were the optimal image resolution in this study.

© 2023 Nagasato D et al. *JAMA Ophthalmology.*

eTable 1. Estimation Accuracy for Mean Deviation Using Ultra-widefield Fundus Autofluorescence (UWFAF) Images With and Without Cropping

DL model	Correlation coefficient	
	UWFAF images with cropping	UWFAF images without cropping
VGG-16	0.672	**0.765**
ResNet50	0.678	**0.751**
InceptionV3	0.744	**0.771**
DenseNet121	0.745	**0.764**
EfficientNetB0	0.606	**0.770**

The estimation accuracy is evaluated using the validation data. The image types that yielded the higher correlation coefficient are indicated in bold red.

Abbreviations: DL, deep learning; ResNet50, Residual Network-50; VGG-16, Visual Geometry Group-16.

© 2023 Nagasato D et al. *JAMA Ophthalmology.*

eTable 2. Estimation Accuracy for Mean Deviation Using Ultra-widefield Fundus Autofluorescence Images With Different Image Resolutions

**DL model**	**Correlation coefficient**	
	**512 × 512-pixel images**	**1024 × 1024-pixel images**
VGG-16	**0.765**	0.660
ResNet50	**0.764**	0.715
InceptionV3	0.771	**0.784**
DenseNet121	**0.751**	0.688
EfficientNetB0	**0.770**	0.527

The estimation accuracy is evaluated using the validation data. The image types that yielded the higher correlation coefficient are indicated in bold red.

Abbreviations: DL, deep learning; ResNet50, Residual Network-50; VGG-16, Visual Geometry Group-16.

© 2023 Nagasato D et al. *JAMA Ophthalmology.*

eTable 3. Mean Absolute Error (MAE) and Root-Mean-Square Error (RMSE)

Parameter, unit	Dataset	MAE	RMSE
MD, dB	All-eyes	5.567	6.918
	AF ring positive	5.839	7.245
	AF ring negative	6.729	8.844
CENT12, dB	All-eyes	4.962	6.540
	AF ring positive	4.771	6.429
	AF ring negative	9.872	11.608
BCVA, logMAR	All-eyes	0.382	0.582
	AF ring positive	0.339	0.506
	AF ring negative	0.518	0.645

MAE and RMSE of the adopted image–model combination are presented.

Abbreviations: AF, autofluorescent; BCVA, best-corrected visual acuity; CENT12, mean sensitivity of central 12 test points on the Humphrey field analyzer; MD, mean deviation on the Humphrey field analyzer.

© 2023 Nagasato D et al. *JAMA Ophthalmology.*

eFigure 1. Cropping of the Ultra-widefield Fundus Autofluorescence Image of the Representative Case

The cropping area is indicated inside the red line. The cropping area is specified as the largest rectangular area containing the optic disc, macula, and fundus, with no surrounding black area. This area extraction task was performed automatically.

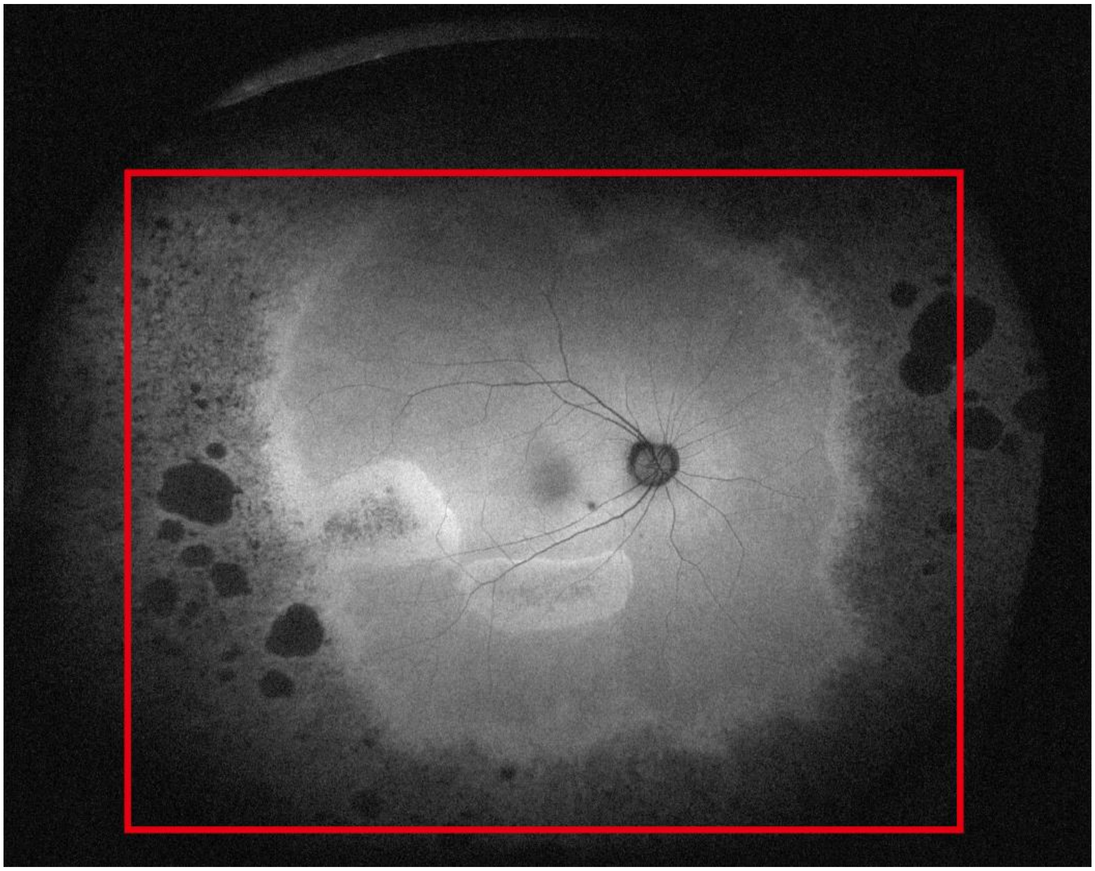

© 2023 Nagasato D et al. *JAMA Ophthalmology.*

eFigure 2. Correlations Between Actual Values and Estimated Values in Groups With the Presence or Absence of AF Rings

Correlations between actual and estimated values of MD (A, D), CENT12 (B, E), and BCVA (C, F) in the groups with the presence or absence of AF rings.

Abbreviations: AF, autofluorescent; BCVA, best-corrected visual acuity; CENT12, mean sensitivity of central 12 test points on the Humphrey field analyzer; MD, mean deviation on the Humphrey field analyzer.

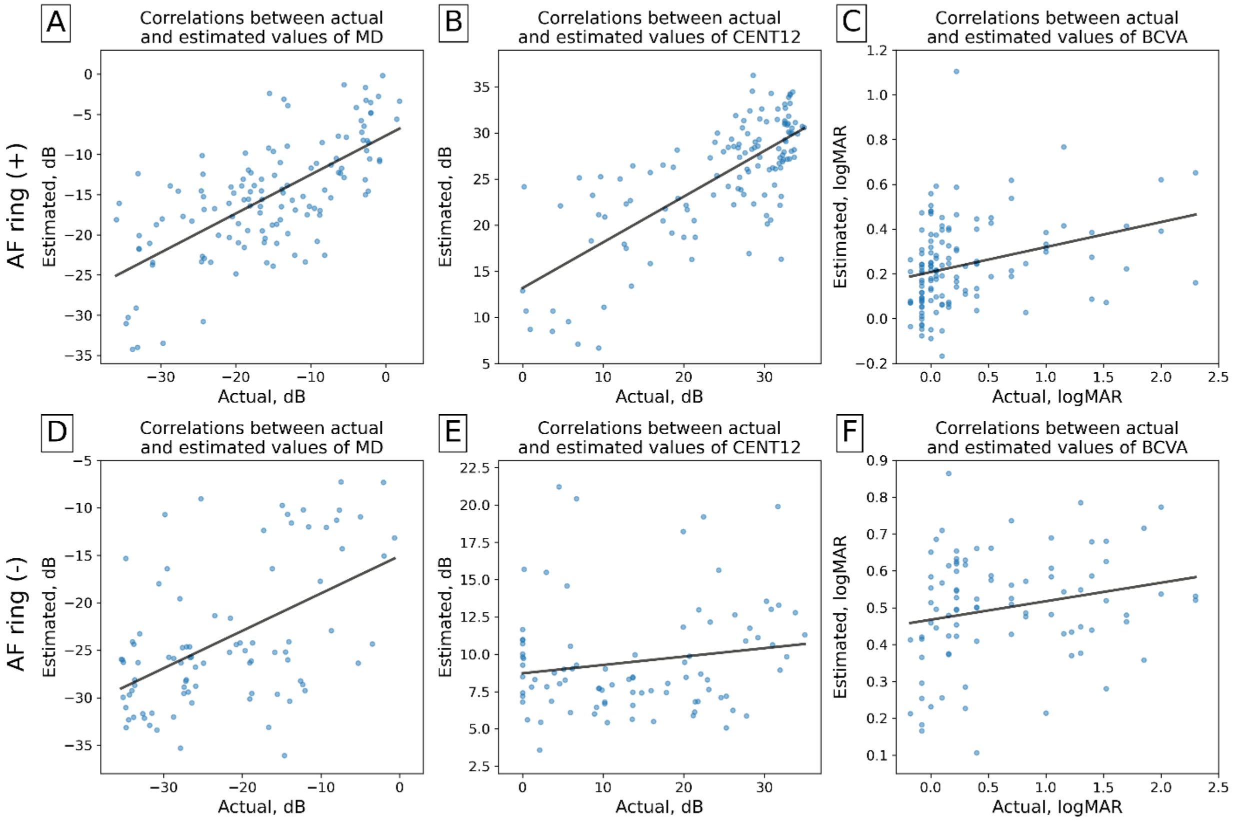

© 2023 Nagasato D et al. *JAMA Ophthalmology.*

eFigure 3. Bland-Altman Plots Between Actual Values and Estimated Values in Groups With the Presence or Absence of AF Rings

Bland-Altman plots between actual and estimated values of MD (A, D), CENT12 (B, E), and BCVA (C, F) in the groups with the presence or absence of AF rings.

In the group with the presence of AF rings, more than 95% of the differences in MD and CENT12 values lay within the limits of agreement. However, proportional bias was prominent in BCVA and existed in MD (correlation coefficient, 0.43; 95% CI, 0.27–0.56; *P* <.001), CENT12 (correlation coefficient, 0.50; 95% CI, 0.36–0.62; *P* <.001), and BCVA (correlation coefficient, 0.79; 95% CI, 0.71–0.84; *P* <.001). There was no fixed bias in MD (mean difference: −0.35; 95% CI, −1.62 to 0.92; *P* =.59), CENT12 (mean difference: −0.74; 95% CI, −1.85 to 0.38; *P* =.20), or BCVA (mean difference: 0.04; 95% CI, −0.05 to 0.12; *P* =.42).

In the group with the absence of AF rings, more than 95% of the differences in CENT12 and BCVA values lay within the limits of agreement. However, proportional bias was prominent in CENT12 and BCVA. Proportional bias existed in MD (correlation coefficient, 0.32; 95% CI, 0.11–0.49; *P* =.002), CENT12 (correlation coefficient, 0.80; 95% CI, 0.71–0.87; *P* <.001), and BCVA (correlation coefficient, 0.90; 95% CI, 0.85–0.94; *P* <.001). There was no fixed bias in MD (mean difference: 1.60; 95% CI, −0.25 to 3.45; *P* =.09) or in BCVA (mean difference: 0.13; 95% CI, 0.00–0.26; *P* =.06), whereas there was a fixed bias in CENT12 (mean difference: 4.47; 95% CI, 2.19–6.75; *P* <.001).
Abbreviations: AF, autofluorescent; BCVA, best-corrected visual acuity; CENT12, mean sensitivity of central 12 test points on the Humphrey field analyzer; MD, mean deviation on the Humphrey field analyzer.

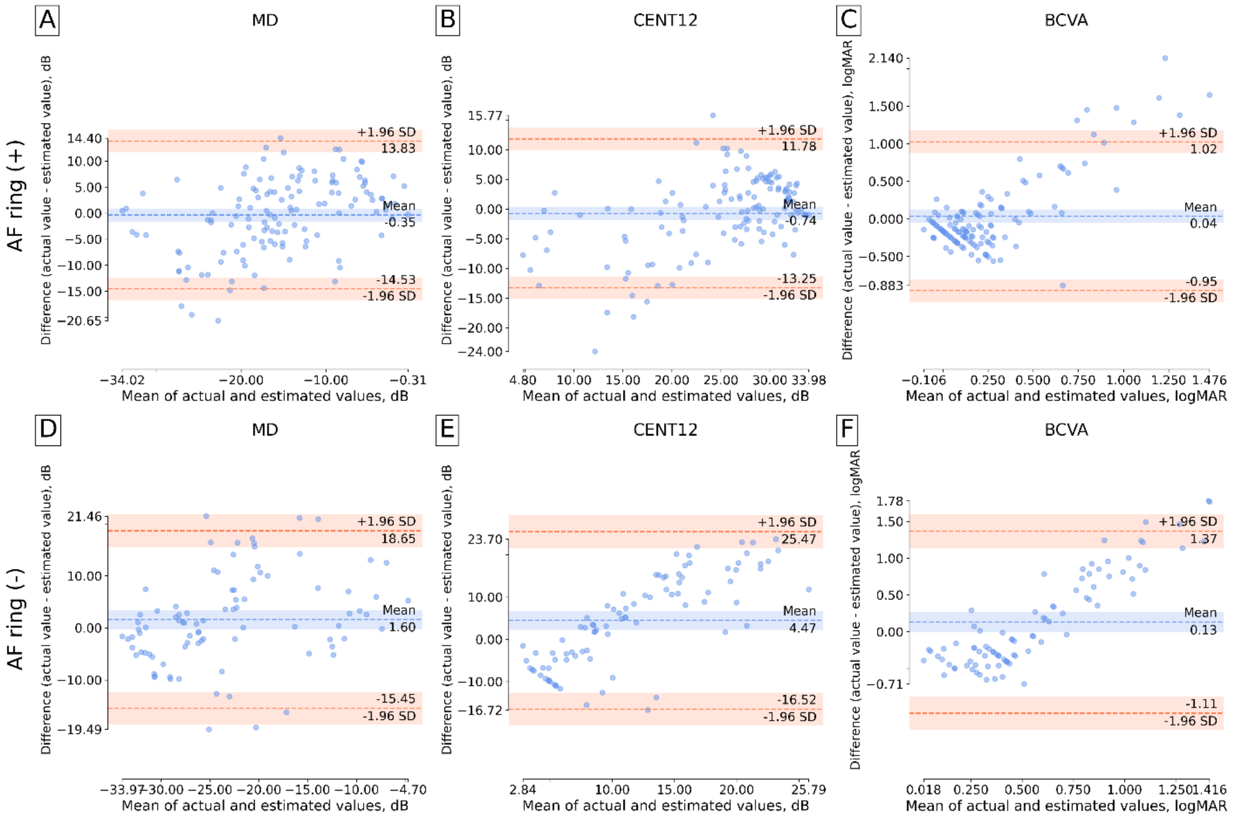

© 2023 Nagasato D et al. *JAMA Ophthalmology.*

eFigure 4. Representative Ultra-widefield Fundus Autofluorescence (UWFAF) Images and Their Heat Map Images

When the mean deviation on the Humphrey field analyzer was estimated using deep learning (DL) models of DenseNet121 (A and B), EfficientNetB0 (C and D), and Residual Network-50 (E and F), the heat map images were superimposed on the UWFAF images in the composite images.

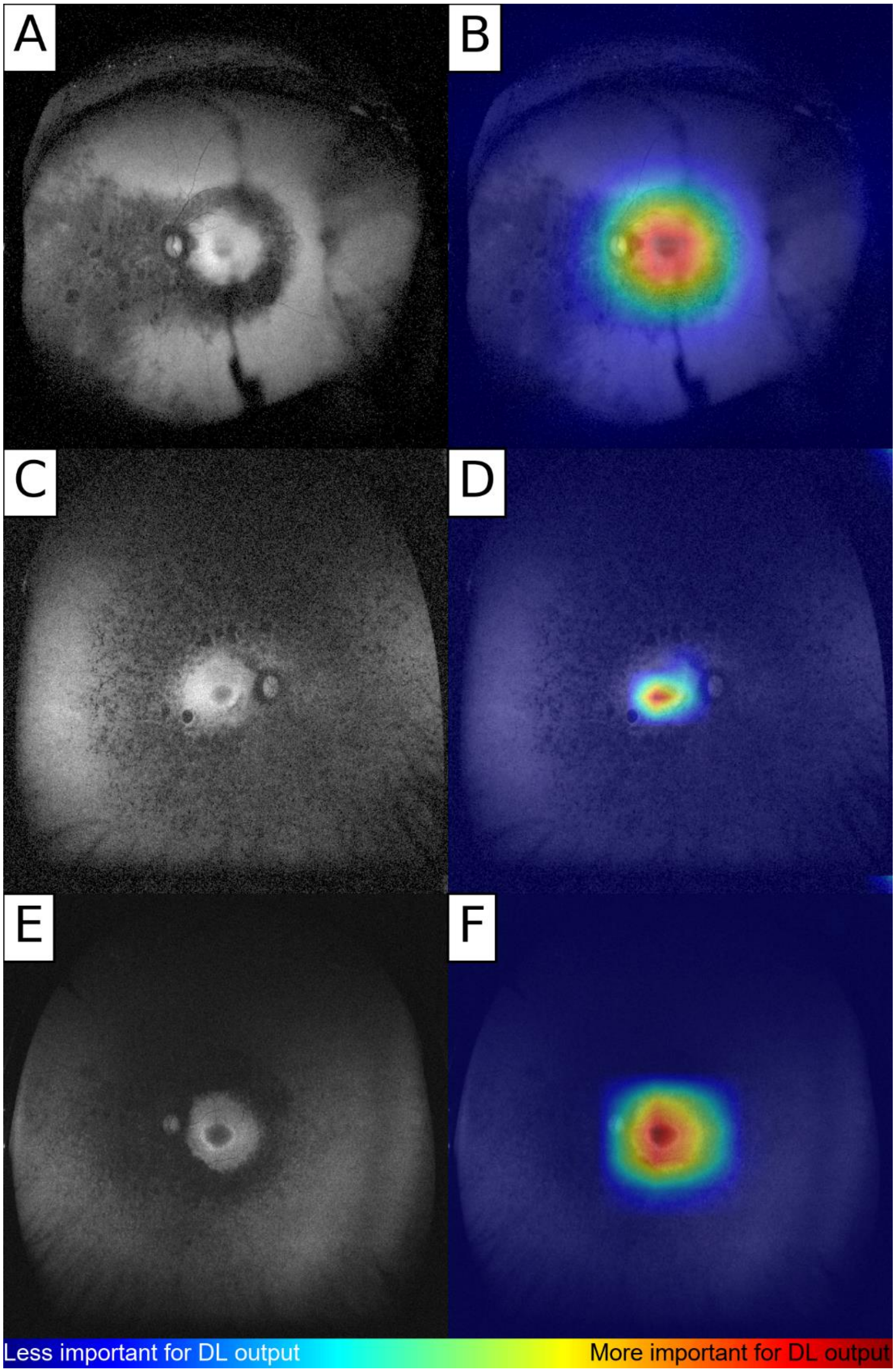

© 2023 Nagasato D et al. *JAMA Ophthalmology.*
