# Supplementary material for: Estimation of Visual Function Using Deep Learning From Ultra-Widefield Fundus Images of Eyes With Retinitis Pigmentosa
Source: JAMA Ophthalmol. 2023 Feb 23;141(4):305–13. doi: 10.1001/jamaophthalmol.2022.6393 (PMC9951103; doi:10.1001/jamaophthalmol.2022.6393)
Supplement: Supplement 2. — Data Sharing Statement [file jamaophthalmol-e226393-s002.pdf]

# Data Sharing Statement

Nagasato. Estimation of Visual Function Using Deep Learning From Ultra-Widefield Fundus Images of Eyes With Retinitis Pigmentosa. *JAMA Ophthalmol*. Published February 23, 2023. doi:10.1001/jamaophthalmol.2022.6393

**Data**
**Data available:** No
